# GLP-1 Receptor Agonists in Chronic Inflammatory Skin Diseases: Immunometabolic Mechanisms and Translational Perspectives

**DOI:** 10.3390/pharmaceutics18050605

**Published:** 2026-05-15

**Authors:** Klara Andrzejczak, Emilia Kucharczyk, Matylda Korgiel, Justyna Drozdowska, Joanna Maj, Małgorzata Ponikowska

**Affiliations:** 1 Faculty of Medicine, Wroclaw Medical University, 50-367 Wroclaw, Poland; klara.andrzejczak@student.umw.edu.pl (K.A.);; 2 University Centre of General Dermatology and Oncodermatology, Wroclaw Medical University, 50-556 Wroclaw, Poland; joanna.maj@umw.edu.pl

**Keywords:** glucagon-like peptide-1 receptor agonists (GLP-1RAs), GLP-1 receptor, immunometabolism, chronic inflammatory skin diseases, psoriasis, atopic dermatitis, hidradenitis suppurativa, inflammation

## Abstract

Chronic inflammatory skin diseases, including psoriasis, hidradenitis suppurativa (HS), and atopic dermatitis (AD), are increasingly recognized as systemic disorders associated with chronic immune dysregulation. Growing evidence supports their links with metabolic disorders, reflected in heightened interest in therapeutic strategies targeting the immunometabolic axis. This review summarizes current knowledge on the role of glucagon-like peptide-1 receptor agonists (GLP-1RAs) in the regulation of immune and metabolic processes in chronic inflammatory skin diseases, with particular emphasis on molecular mechanisms and available experimental and clinical data. GLP-1RAs, widely used in the treatment of type 2 diabetes and obesity, may also exhibit anti-inflammatory and immunomodulatory properties beyond their classical metabolic effects. GLP-1 signalling can influence keratinocyte function, immune cell activity, and wound healing. Furthermore, it modulates multiple intracellular signalling pathways, including cAMP/PKA, AMPK, PI3K/Akt, and NF-κB, as well as immune axes such as IL-23/Th17/IL-17 and inflammasome-related signalling. Available evidence suggests that GLP-1RAs may reduce inflammation and disease activity in selected inflammatory dermatoses. However, current evidence remains limited and is based primarily on experimental studies, case reports, and small-scale observational studies. Further well-designed clinical trials are needed to better define the therapeutic potential of GLP-1RAs and their role in dermatological practice.

## 1. Introduction

Immune-mediated inflammatory diseases (IMIDs), including psoriasis, hidradenitis suppurativa (HS), and atopic dermatitis (AD), represent a heterogeneous group of disorders arising from dysregulation of complex immune mechanisms. They are increasingly recognized as systemic diseases rather than conditions confined to the skin, reflecting chronic immune activation and elevated levels of circulating proinflammatory mediators.

In this context, these conditions can be viewed as immunometabolic disorders, highlighting the close interplay between immune dysregulation and metabolic disturbances such as obesity, insulin resistance (IR), and metabolic syndrome. These relationships are particularly pronounced in psoriasis and HS, while they remain less clear in AD, although recent studies indicate a growing body of evidence linking this condition to metabolic disorders [1,2,3,4,5].

Glucagon-like peptide-1 (GLP-1) is an incretin hormone produced in the intestine and released in response to food intake, playing a key role in regulating glucose homeostasis by stimulating insulin secretion and inhibiting glucagon secretion. It also regulates appetite by delaying gastric emptying and enhancing satiety, thereby contributing to improved glycaemic control and weight reduction [6,7].

The broad tissue expression of glucagon-like peptide-1 receptor (GLP-1R) suggests that the effects of GLP-1 extend beyond its classical metabolic roles. Available data indicate that GLP-1R signalling influences both skin cells, including keratinocytes, and immune cells present within inflammatory lesions. In chronic inflammatory skin diseases, GLP-1R expression may partially reflect immune cell infiltration, suggesting a role for this pathway in the regulation of inflammatory processes, cell migration, and skin repair mechanisms [8,9,10,11]. Furthermore, keratinocytes may represent a direct target of GLP-1R signalling [12].

The growing importance of the immunometabolic axis in the pathogenesis of these diseases has led to increased interest in glucagon-like peptide-1 receptor agonists (GLP-1RAs), which are widely used for the treatment of type 2 diabetes mellitus (T2DM) and obesity, as well as for cardiovascular risk reduction. In addition to their well-documented metabolic effects, they exhibit pleiotropic anti-inflammatory and immunomodulatory properties that extend beyond glycaemic control and weight loss, highlighting their potential as therapeutic agents in chronic inflammatory skin diseases. This is particularly relevant in the context of coexisting metabolic disorders, such as obesity, IR, and chronic inflammation, which contribute to the development and exacerbation of these diseases [7,13,14,15,16].

Obesity plays an important role in modulating the inflammatory response. Adipose tissue functions as an active immunoregulatory organ, exacerbating inflammation through increased secretion of leptin and proinflammatory cytokines. This contributes to increased disease activity, a higher burden of comorbidities, and reduced treatment efficacy, providing a rationale for therapeutic approaches targeting metabolic pathways [17,18,19].

This review provides a comprehensive overview of GLP-1RAs as immunometabolic therapies in chronic inflammatory skin diseases. Attention is paid to their mechanisms of action, their role in modulating immune and metabolic processes, available clinical data, and translational implications. The interplay between metabolic and inflammatory mechanisms in these diseases suggests that targeting the immunometabolic axis may represent a promising therapeutic strategy, potentially as an adjunct to existing treatments.

## 2. Brief Mechanism of Action GLP-1

GLP-1 is a 36-amino-acid incretin hormone produced in enteroendocrine L-cells located mainly in the distal small intestine and colon. It exists in two biologically active forms, GLP-1(7–36) amide and GLP-1(7–37), which are equipotent, although GLP-1(7–36) amide represents the predominant physiological form in humans [20,21]. GLP-1 is considered the second incretin hormone identified after gastric inhibitory peptide (GIP), which was originally isolated from porcine small intestine and shown to inhibit gastric acid secretion [22].

**Figure 1:**
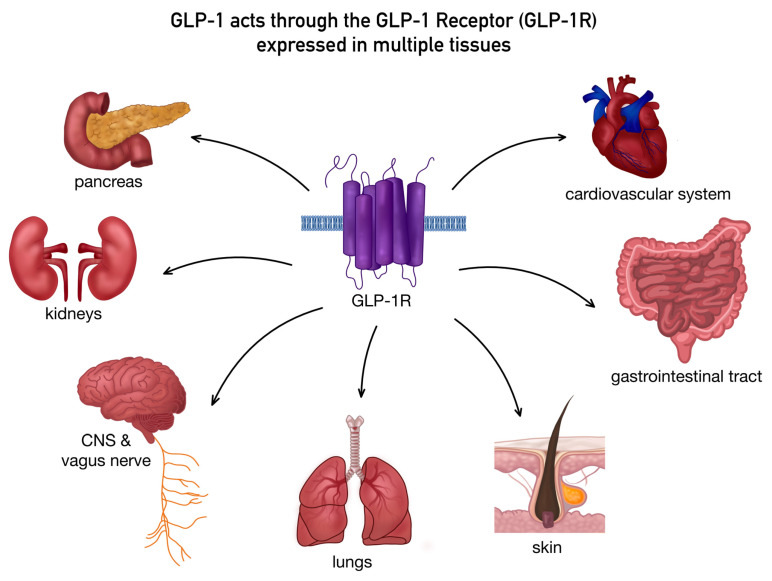
Tissue Distribution of the GLP-1 Receptor (GLP-1R) [11,24,25,26,27]. Abbreviations: GLP-1—Glucagon-like peptide-1; GLP-1R—GLP-1 receptor; CNS—central nervous system.

The physiological half-life of endogenous GLP-1 is extremely short, typically around 1–2 min [23], mainly due to rapid degradation by the enzyme dipeptidyl-peptidase-4 (DPP-4), which converts GLP-1 into the metabolite GLP-1(9–36) amide (the natural DPP4 cleavage product), as well as through renal clearance [20,22]. GLP-1 mediates its biological effects through the GLP-1R, a member of the class B family of G-protein-coupled receptors that is expressed in multiple tissues including the pancreas, lungs, kidneys, cardiovascular system, gastrointestinal tract, central nervous system, vagus nerve, and skin [11,24,25,26,27]. Figure 1 illustrates the widespread expression of the GLP-1R across multiple tissues, emphasizing the diverse physiological systems through which GLP-1 mediates its biological effects.

In addition, GLP-1R activation has been shown to inhibit abnormal vascular smooth muscle cell proliferation through modulation of the PI3K/AKT and ERK1/2 signalling pathways [28].

In pancreatic β-cells, GLP-1 binding to GLP-1R activates adenylate cyclase, leading to an increase in intracellular cyclic adenosine monophosphate (cAMP). Elevated cAMP subsequently activates protein kinase A (PKA) and the cAMP-regulated guanine nucleotide exchange factor Epac2 (also known as cAMP-GEF2) [29]. The activation of PKA causes the closure of ATP-sensitive potassium channels, which results in membrane depolarization and the opening of L-type voltage-dependent calcium channels, thereby leading to calcium influx and the production of action potentials [30,31]. PKA signalling also promotes calcium release through ryanodine receptors (RyR) and inositol-1,4,5-trisphosphate (IP3) pathways, further increasing intracellular Ca^2+^ levels. In parallel, Epac2 activates Rap1 and phospholipase C signalling, resulting in additional IP3 and diacylglycerol production and further calcium mobilization [29,32,33]. The overall effect of all these signalling cascades is the increase in the intracellular Ca^2+^ concentration, which enhances the production of ATP in the mitochondria and the exocytotic release of insulin from the pancreatic β-cells [34].

Due to the rapid degradation of the natural GLP-1, much pharmacological effort has been directed towards the development of GLP-1RAs, which have been used extensively in the treatment of T2DM. Based on the structural characteristics, the GLP-1RAs have been divided into two categories: GLP-1 analogues and exendin-4 analogues [35,36]. Examples include exenatide and liraglutide, both of which are resistant to DPP-4 degradation and provide prolonged receptor activation.

Metabolic diseases such as obesity, IR, and systemic inflammation are strongly associated with several chronic inflammatory skin diseases [8,9,20]. Consequently, the metabolic and immunomodulatory properties of GLP-1 receptor agonists have attracted increasing interest in dermatology, suggesting a potential therapeutic role in conditions where metabolic dysregulation and cutaneous inflammation intersect [8].

## 3. GLP-1 Receptor Signalling and Cellular Effects

### 3.1. GLP-1R Expression and Direct Cutaneous Effects

**Figure 2:**
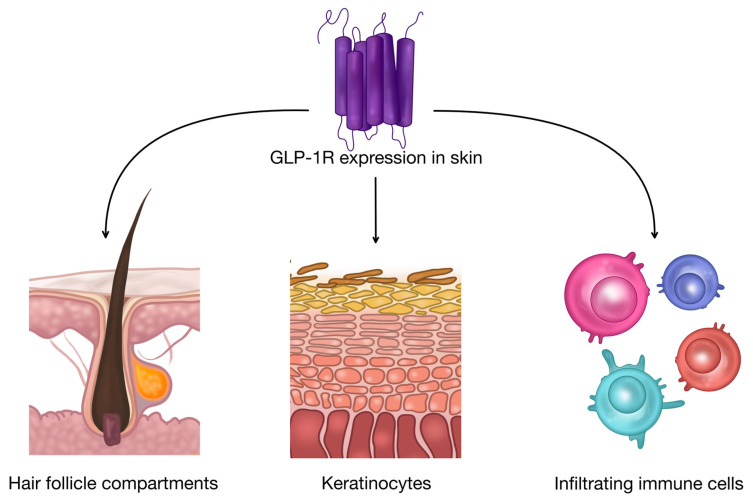
GLP-1 Receptor (GLP-1R) Expression in Skin: Keratinocytes, Hair Follicle Compartments, and Infiltrating Immune Cells [10,11,37,38].

Emerging evidence suggests that GLP-1R is expressed in several components of the skin, as illustrated in Figure 2, indicating that GLP-1 signalling may exert direct local effects within the cutaneous microenvironment [10,11,37,38].

Experimental studies investigating GLP-1R distribution in murine skin demonstrated receptor expression using RT-PCR, Western blotting, and in situ immunocytochemistry, with localization predominantly within specific regions of hair follicles [39]. In the same study, proglucagon expression was also detected in skin tissue, and GLP-1 stimulation activated MAPK/ERK signalling in cultured skin-derived cells, suggesting the existence of a local GLP-1 signalling axis in the skin [39,40]. Mechanistically, GLP-1 has also been shown to transactivate the epidermal growth factor receptor (EGFR), potentially via c-Src activation and the release of endogenous EGF-like ligands, linking GLP-1 signalling with pathways involved in epithelial growth and tissue remodelling [41,42]. Additionally, cultured skin-derived cells exposed to GLP-1 express the neural stem cell marker nestin, a feature associated with dedifferentiated epithelial cells and epithelial–mesenchymal transition processes [43,44]. Together, these observations support the hypothesis that GLP-1 may function through autocrine or paracrine mechanisms regulating skin development and folliculogenesis [39].

Further evidence indicates that keratinocytes themselves may represent functional targets of GLP-1 receptor signalling. In cultured keratinocytes, GLP-1R mRNA and protein expression have been confirmed, and stimulation with the GLP-1 analogue liraglutide was shown to enhance keratinocyte migration through activation of the PI3K/Akt pathway. In vivo experiments in murine models further demonstrated that liraglutide administration facilitated wound healing, supporting a direct role for GLP-1R signalling in epidermal repair processes [12]. Activation of the GLP-1R/PI3K/Akt pathway has also been related to cytoprotective effects in other tissues, which include the inhibition of cell death programs such as necroptosis, and therefore it is possible that similar pathways could be involved in the increased cell survival and regeneration observed during cutaneous wound healing [45]. Moreover, the activation of the PI3K/Akt/mTOR pathway, which is a downstream target of the GLP-1R pathway, has been correlated with increased cell proliferation and differentiation. This implies a broader role for the GLP-1R pathway in the context of tissue remodelling and repair, which is important in the context of wound healing [46]. Additionally, the activation of the GLP-1R pathway has been correlated with the normalization of abnormal cell migration, proliferation, and apoptosis under stressful conditions via the modulation of the PI3K/Akt and ERK1/2 pathways. This implies a broader role for the GLP-1R pathway in the restoration of normal cellular homeostasis in the context of wound healing [28].

GLP-1R expression has also been observed in immune cells infiltrating the skin, which may be particularly relevant in inflammatory dermatoses. In a study in which skin biopsies of patients with plaque psoriasis were examined, the expression of GLP-1R was observed in five of six affected skin samples, whereas it was only rarely observed in unaffected psoriatic skin or in healthy skin [9]. Importantly, cultured keratinocytes stimulated with pro-inflammatory cytokines such as tumour necrosis factor-α (TNF-α) and interferon-γ (IFN-γ) did not demonstrate GLP-1R expression, suggesting that receptor detection in psoriatic plaques likely reflects infiltration by GLP-1R-expressing immune cells rather than constitutive expression by keratinocytes [47,48]. These findings highlight a potential immunomodulatory role of GLP-1 signalling within inflamed skin.

Clinical observations further support the possibility of direct cutaneous actions of GLP-1RAs. A case report described by Faurschou et al. marked improvement of long-standing psoriasis shortly after initiation of liraglutide therapy for T2DM, with reduction in scaling and pruritus occurring before significant weight loss or metabolic improvement, suggesting a potential direct anti-inflammatory effect of GLP-1R activation in the skin [26,49,50,51,52].

### 3.2. Key Anti-Inflammatory Signaling Pathways

Beyond their metabolic actions, GLP-1RAs exert important immunomodulatory and anti-inflammatory effects through several intracellular signalling pathways relevant to inflammatory skin diseases. One key mechanism involves modulation of innate immune cells such as invariant natural killer T (iNKT) cells, which play an important role in psoriasis pathogenesis [53]. iNKT cells recognize lipid antigens presented by CD1d molecules and rapidly produce cytokines including IFN-γ, IL-4, IL-17, TNF-α, and GM-CSF, thereby shaping inflammatory responses in the skin [54,55].

GLP-1 receptors are expressed on iNKT cells, and stimulation with GLP-1 or its analogues can modulate their activity and distribution [52]. In patients with psoriasis treated with GLP-1RAs, circulating iNKT cell numbers increase while their accumulation within psoriatic plaques decreases, suggesting redistribution of these cells away from inflamed skin [26,52].

At the molecular level, GLP-1R activation stimulates adenylate cyclase through G-protein coupling, leading to increased intracellular cAMP and activation of PKA. PKA subsequently phosphorylates the transcription factor cAMP response element-binding protein (CREB) [56]. CREB activation can inhibit the NF-κB signalling pathway and suppress transcription of pro-inflammatory mediators, thereby attenuating inflammatory cytokine production and potentially promoting anti-inflammatory responses such as IL-10 induction [26,56,57].

Anti-inflammatory effects of GLP-1RAs have also been demonstrated in skin-resident cells. In lipopolysaccharide-stimulated HaCaT keratinocytes, liraglutide reduced activation of inflammatory pathways including NF-κB, JAK2/STAT3, and SOCS3 signalling while decreasing secretion of pro-inflammatory cytokines such as TNF-α and IL-6 [38,58]. These effects were mediated through activation of AMP-activated protein kinase (AMPK), as pharmacological inhibition of AMPK reversed the anti-inflammatory activity of liraglutide [38].

Evidence from tissue injury models further supports cytoprotective and anti-inflammatory actions of GLP-1RAs in the skin. In a murine random-pattern skin flap model, the GLP-1 analogue exenatide significantly improved flap survival by enhancing angiogenesis, reducing oxidative stress, and attenuating pyroptosis. These effects were mediated through activation of autophagy via TFE3 nuclear translocation and AMPK-dependent pathways, including AMPK–SKP2–CARM1 and AMPK–mTOR signalling [59]. The consistent anti-inflammatory and anti-angiogenic effects have also been reported in ocular inflammatory models. Liraglutide was found to reduce pathological angiogenesis and the expression of inflammatory mediators, including NLRP3 inflammasome, IL-1β, IL-6, and TNF-α, in a laser-induced choroidal neovascularization model [60]. These findings suggest that GLP-1R signalling inhibits inflammatory pathways through cAMP/PKA pathways in immune cells and through the regulation of inflammatory pathways by AMPK in keratinocytes [58,60].

**Figure 3:**
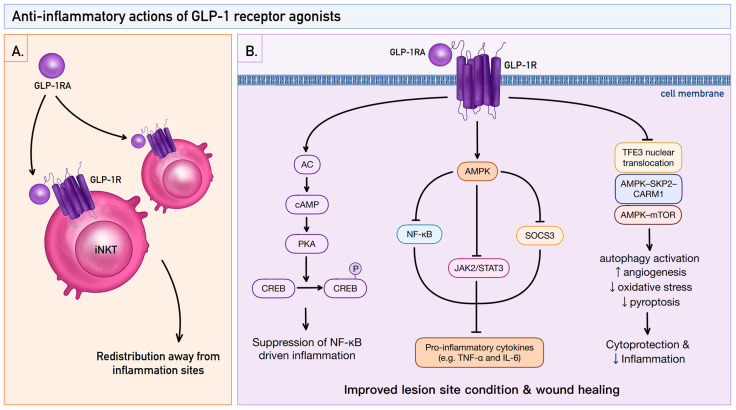
(**A**,**B**) Anti-inflammatory mechanisms of GLP-1 receptor agonists (GLP-1RAs) in immune and skin cells. GLP-1R activation induces anti-inflammatory effects via cAMP/PKA/CREB-mediated inhibition of NF-κB and reduced cytokine production. In keratinocytes, GLP-1RAs suppress NF-κB, JAK2/STAT3, and SOCS3 signalling through AMPK activation. Additionally, AMPK-dependent pathways promote autophagy, angiogenesis, and cytoprotection, leading to reduced oxidative stress, pyroptosis, and overall inflammation [26,38,45,56,57,58,59,60].

Together, these mechanisms highlight the multifaceted anti-inflammatory actions of GLP-1R activation across both immune and skin-resident cells, supporting its potential relevance in chronic inflammatory dermatoses (Figure 3).

## 4. Modulation of Immunology

### 4.1. Modulation of the IL-23/Th17/IL-17 Axis in Inflammatory Skin Disease

The IL-23/Th17/IL-17 axis represents a central inflammatory pathway in several chronic skin diseases, particularly psoriasis. IL-17 acts as a key effector cytokine that promotes keratinocyte activation, neutrophil recruitment, and amplification of inflammatory signalling within the skin [61,62].

Evidence suggests that GLP-1RAs may influence this pathway through modulation of IL-17-producing immune cells. Dermal γδ T cells constitute an important source of IL-17 in psoriatic lesions and play a significant role in disease pathogenesis [37]. Treatment with GLP-1 analogues such as exenatide or liraglutide has been associated with reduced numbers of dermal γδ T cells and decreased IL-17 expression in psoriatic skin, accompanied by improvement in clinical disease severity [50]. Furthermore, GLP-1 signalling may inhibit TNF-α–driven inflammatory cascades and reduce lymphocyte recruitment by decreasing stromal cell-derived factor-1 (SDF-1)-mediated chemotaxis [50,63,64]. In a prospective case-series study, reductions in Psoriasis Area and Severity Index (PASI) scores correlated with decreases in dermal γδ T-cell percentages and IL-17 expression following GLP-1 analogue therapy, suggesting that modulation of IL-17-producing immune populations may contribute to the observed clinical effects [50].

### 4.2. Microbial-Driven Inflammation and LPS Signalling

Microbial dysbiosis has emerged as a key mediator of inflammation in AD. Patients suffering from AD have low microbial diversity as well as increased microbial colonization, which is strongly correlated with disease severity as well as exacerbations [65]. Bacterial components such as lipopolysaccharide (LPS) act as potent activators of innate immune responses by triggering inflammatory signalling pathways including NF-κB activation in macrophages and other immune cells [66].

Experimental models of AD demonstrate that LPS-driven inflammatory signalling contributes to increased production of nitric oxide and pro-inflammatory mediators through activation of NF-κB and MAPK pathways. In macrophage models, LPS stimulation induces overexpression of inflammatory mediators such as inducible nitric oxide synthase (iNOS) and cyclooxygenase-2, further amplifying cutaneous inflammation [67,68].

These microbial-driven inflammatory pathways are particularly relevant in AD, where impaired skin barrier function facilitates microbial colonization and promotes chronic immune activation. As GLP-1R signalling has been shown to suppress key inflammatory pathways such as NF-κB and MAPK signalling in immune and skin-resident cells, modulation of LPS-induced inflammatory responses may represent another mechanism through which GLP-1RAs could influence inflammatory skin diseases [38].

### 4.3. GLP-1 Receptor Agonists and Immunometabolic Regulation of Wound Healing

Accumulating evidence suggests that GLP-1RAs exert pro-regenerative effects in cutaneous wound healing through coordinated modulation of immune responses, angiogenesis, and keratinocyte function. Chronic wounds are characterized by persistent inflammation, impaired macrophage polarization, defective angiogenesis, and delayed re-epithelialization [69]. GLP-1RA signalling appears to address several of these pathophysiological barriers by regulating inflammatory pathways and promoting tissue repair processes [10].

One important mechanism involves direct stimulation of keratinocyte migration and proliferation. Experimental studies have demonstrated that keratinocytes express GLP-1Rs, and activation of this pathway by liraglutide significantly enhances keratinocyte migration through activation of the PI3K/Akt signalling pathway, thereby accelerating re-epithelialization in experimental wound models [12]. More recent mechanistic studies further revealed that liraglutide can regulate cytoskeletal and adhesion signalling in keratinocytes through stabilization of unconventional myosin-1c (Myo1c), which enhances interaction with dedicator of cytokinesis-5 (Dock5). Activation of the Myo1c/Dock5 axis promotes keratinocyte proliferation, migration, and adhesion, ultimately improving collagen deposition, extracellular matrix remodelling, and wound closure in diabetic mouse models [70].

GLP-1RA-mediated wound repair is also closely linked to modulation of innate immune responses. Experimental studies demonstrate that liraglutide promotes polarization of macrophages from pro-inflammatory M1 phenotypes toward reparative M2 macrophages during wound healing [71]. This shift enhances the production of pro-repair mediators and angiogenic factors, including vascular endothelial growth factor (VEGF), thereby promoting neovascularization and tissue regeneration [7,71,72]. Additional immunometabolic mechanisms involve the regulation of oxidative stress and cell death pathways. In diabetic wound models, dulaglutide has been shown to activate the Nrf2 antioxidant signalling pathway, increasing expression of glutathione peroxidase (GPX4) and SLC7A11 while reducing lipid peroxidation and ferroptosis. These effects restore redox balance and improve keratinocyte viability, contributing to enhanced wound closure [73].

In addition, the activation of the GLP-1R has been shown to stimulate angiogenesis and endothelial repair. In diabetic animal models, the administration of the GLP-1RA, exendin-4, has been shown to increase the number of endothelial progenitor cells and the expression of vascular remodelling mediators, such as VEGFR-2, phosphorylated endothelial nitric oxide synthase, transforming growth factor-beta, and matrix metalloproteinase-2. These processes play critical roles in the proliferative and remodelling phases of wound healing [74,75]. The GLP-1RA-based approach may be beneficial for the enhancement of antimicrobial defense and vascularization in infected wounds. Biomaterial-based delivery systems that combine liraglutide and zinc oxide nanoparticles have been shown to have synergistic antibacterial activities against *Staphylococcus aureus* and *Escherichia coli*, and promote endothelial cell migration, tubule formation, and wound healing [76,77].

These studies indicate that GLP-1RAs enhance wound healing through multiple complementary mechanisms, including activation of PI3K/Akt signalling in keratinocytes, regulation of cytoskeletal migration pathways, modulation of macrophage polarization, suppression of oxidative stress and ferroptosis, and stimulation of angiogenesis [10,12,28]. These pleiotropic immunometabolic effects suggest that GLP-1R signalling may represent a promising therapeutic target for chronic and diabetes-associated cutaneous wounds.

**Figure 4:**
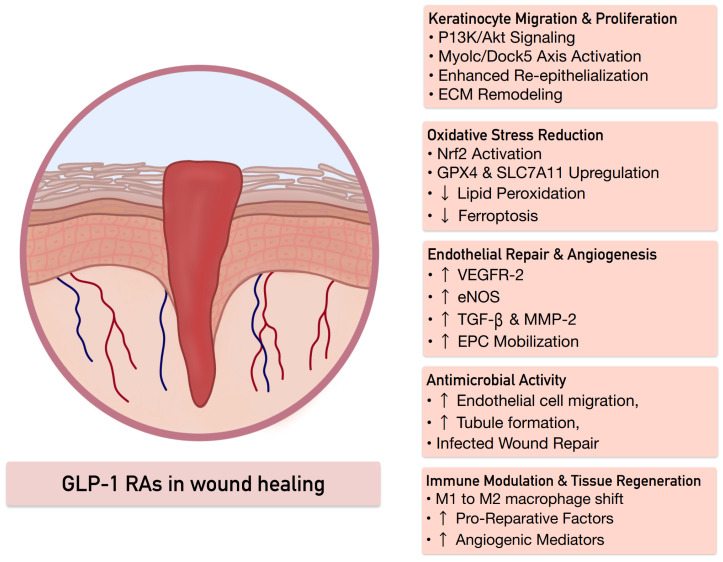
Multimodal Mechanisms of GLP-1 Receptor Agonists (GLP-1Ras) in Cutaneous Wound Healing. GLP-1RAs promote wound repair by enhancing keratinocyte migration and proliferation, reducing oxidative stress via Nrf2 activation, and inhibiting ferroptosis. They stimulate angiogenesis and endothelial repair, modulate immune responses through M1 to M2 macrophage polarization, and exhibit antimicrobial effects. Together, these actions improve vascularization, tissue regeneration, and wound closure [10,12,28,70,71,72,73,74,76,77,78,79]. Abbreviations: GLP-1RA—glucagon-like peptide-1 receptor agonist; PI3K—phosphoinositide 3-kinase; Akt—protein kinase B; Myo1c—unconventional myosin-1c; Dock5—dedicator of cytokinesis 5; ECM—extracellular matrix; Nrf2—nuclear factor erythroid 2-related factor 2; GPX4—glutathione peroxidase; SLC7A11—solute carrier family 7 member 11; VEGFR-2—vascular endothelial growth factor receptor 2; eNOS—endothelial nitric oxide synthase; TGF-β—transforming growth factor beta; MMP-2—matrix metalloproteinase-2; EPC—endothelial progenitor cell; M1—classically activated (pro-inflammatory) macrophage phenotype; M2—alternatively activated (pro-reparative) macrophage phenotype.

The diverse mechanisms through which GLP-1RAs promote wound healing including keratinocyte activation, immune modulation, reduction in oxidative stress, stimulation of angiogenesis, and antimicrobial effects are summarized in Figure 4 [10,12,28,70,71,72,73,74,76,77,78,79].

## 5. Clinical Evidence in Chronic Inflammatory Skin Diseases

### 5.1. Psoriasis

Modern medicine is redefining psoriasis not merely as an isolated cutaneous disorder, but as a critical component of a broader metabolic “comorbidome”. This shift necessitates an evolution in therapeutic strategies toward agents capable of modulating systemic inflammation. At the core of this relationship lies a bidirectional link between psoriasis and T2DM, where chronic immune activation drives escalating IR, leading to progressive vascular damage [26,80,81]. Genetic studies further highlight a shared pathogenic foundation, identifying susceptibility genes such as *PTPN22*, *ST6GAL1*, and *JAZF1*, which participate in inflammatory pathways mediated by T-cells [82].

Population-based analyses utilizing the AoUDB and TriNetX databases demonstrate that the use of GLP-1RAs is associated with a reduced risk (approximately 40–60%) of developing psoriasis and psoriatic arthritis (PsA) [5,83]. This effect has been reported as statistically significant in observational datasets, regardless of the degree of weight loss or the patient’s glycaemic status, although causal relationships cannot be firmly established, pointing toward a potential extra-metabolic immunomodulatory action of these molecules [83]. Furthermore, recent biomarker research indicates that incretin dysregulation and shifts in GIP-1 and leptin levels manifest in the serum long before the onset of clinical joint symptoms, suggesting that GLP-1RAs may represent a potential tool to influence the ‘’psoriatic march” [84]. The clinical response to GLP-1 RA therapy has been reported as relatively rapid in some cases. Patients may report a substantial reduction in pruritus and improved sleep quality within the first 48 h of administration, significantly predating any measurable changes in body mass. Although these observations are primarily derived from limited clinical reports [26]. Direct evidence of these phenomena is found in the case of a 59-year-old male with T2DM [49], where the initiation of liraglutide triggered an immediate dampening of cutaneous inflammation (PGA score drop from 3 to 1) despite a baseline Body Mass Index (BMI) of 29.3 kg/m^2^. The fact that previous glycaemic control had been intermittently achieved without skin improvement may suggests that the drug’s direct anti-inflammatory properties, rather than simple metabolic stabilization, initiated the healing process [49]. These findings are supported by small prospective data, including a cohort study in which T2DM patients saw their mean PASI score drop from 15.7 to 2.0 after 12 weeks of liraglutide therapy [82]. A unique contribution of this study is the histopathological evidence: skin biopsies revealed reduced epidermal thickness, the total disappearance of Munro’s microabscesses, and the resolution of neutrophilic infiltrates, objectively suggesting the drug’s potent anti-inflammatory action within the skin tissue itself. Concurrently, significant improvements in pancreatic function and a reduction in IR, measured by the HOMA-IR index, were observed [82]. However, results from the first randomized placebo-controlled trial (RCT) shed new light on this drug class in obese patients without diabetes. Within a short 8-week observation period window, liraglutide induced a 2.6-point drop in PASI, which did not reach statistical significance compared to placebo. This suggests that in normoglycemic individuals, immunomodulatory mechanisms may require longer exposure times or more potent molecules, such as semaglutide, to overcome epidermal tissue resistance [85].

A breakthrough came with a 2024 cohort study involving 43 patients treated with semaglutide (1.6 mg/week) over six months [86]. The study demonstrated a significant reduction in PASI and skin pain (VAS pain). Utilizing nutritional ultrasound, researchers proved that dermatological improvement correlates with the selective reduction in metabolically active preperitoneal fat. Crucially, the impact on PASI, Dermatology Life Quality Index (DLQI), and mood (BDI scale) remained statistically significant even after adjusting for percentage weight loss, confirming a mechanism of action independent of weight reduction, although these findings require confirmation in larger controlled trials [86].

Further evidence is provided by clinical cases of patients with atypical metabolic profiles or resistance to standard care. One report described a non-obese male (BMI 25.5 kg/m^2^) whose psoriasis went into remission following exenatide therapy; a “re-challenge” effect (relapse after discontinuation and improvement upon reintroduction) supports a possible association [87]. Similarly, the case of a 73-year-old patient with morbid obesity showed that semaglutide may represent an alternative therapeutic option even after the failure of biological therapy (adalimumab), leading to a PASI reduction of over 92% [88]. The molecular model of GLP-1RA action is based on precise intracellular regulation. These agents activate the cAMP/CREB pathway and stimulate AMPK kinase in the epidermis, leading to the suppression of critical inflammatory pathways, including NF-κB and the JAK2/STAT3 axis [84]. Inhibiting STAT3 phosphorylation may contribute to limiting the pathological hyperproliferation of keratinocytes and dampens their ability to recruit macrophages, effectively silencing psoriatic plaque activity [38,88]. A vital link in this response is the effect on iNKT cells. In psoriatic patients, the number of circulating iNKT cells is naturally diminished as they sequester in the skin. GLP-1RA therapy has been shown to increase circulating iNKT levels by nearly 38%, suggesting a migration from inflammatory tissues back into the systemic circulation [52,82]. It has even been hypothesized that the dramatic improvement of psoriasis following bariatric surgery is largely due to the postoperative spike in endogenous GLP-1 levels modulating these specific cells [87].

The drug’s target was confirmed by the detection of GLP-1R mRNA directly in biopsies of active psoriatic plaques, while it was absent in healthy skin [9]. This suggests that the primary therapeutic target within the skin is the immune infiltrate. Although a recent epidemiological analysis [89] indicated a rare 17% increase in the risk of de novo psoriasis (possibly due to gut microbiota modulation), systemic anti-inflammatory resolution- evidenced by falling levels of CRP, ferritin, and homocysteine- remains the dominant clinical outcome of therapy [80,86,89]. Despite the overwhelming evidence of therapeutic efficacy, the first case of paradoxical reaction recently appeared in the literature [90]. It concerns a 34-year-old female patient who experienced a sudden exacerbation of skin lesions only two weeks after starting liraglutide. The authors propose a “cytokine imbalance” hypothesis, suggesting that in rare individuals, the rapid inhibition of the IL-23/IL-17 axis may lead to a compensatory surge of interferon-alpha (IFN-α). While isolated, this report serves as a reminder of the need for individualized patient monitoring, particularly in those predisposed to volatile immune responses [90].

In conclusion, current evidence suggests that GLP-1RAs may represent a promising adjunctive approach in metabolic dermatology, particularly in patients with coexisting metabolic disorders. While available data indicate potential benefits in reducing psoriatic disease activity, improving DLQI, and modulating systemic inflammation, the overall level of clinical evidence remains limited and is largely based on case reports, small observational studies, and relatively short-term trials. Therefore, well-designed, large-scale randomized controlled studies are needed to confirm the magnitude, consistency, and long-term clinical relevance of these effects [5,9,15,38,49,52,82,83,84,85,86,87,88,90,91].

Table 1 summarizes the current clinical and mechanistic evidence regarding the use of GLP-1RAs in psoriasis. It includes data from clinical trials, cohort studies, and case reports, highlighting their effects on disease severity (PASI), DLQI, metabolic parameters, and key inflammatory pathways.

### 5.2. Hidradenitis Suppurativa (HS)

HS is a chronic inflammatory dermatosis in which pilosebaceous unit dysfunction plays a pivotal role in the underlying pathogenesis [93]. The condition is clinically characterized by the presence of painful nodules, abscesses, and fistulae, predominantly localized within intertriginous areas and regions abundant in apocrine glands, such as the axillae and inguinal folds [94]. This disorder exhibits a robust correlation with metabolic disturbances; an excess of adipose tissue triggers a decline in anti-inflammatory adiponectin levels while simultaneously serving as a primary source for the overproduction of pro-inflammatory adipokines and cytokines, including IL-1β, TNF- α, and IL-6 [94,95]. The systemic circulation of these mediators not only exacerbates the dermatological progression but also induces systemic IR and a proatherogenic state [93].

Considering these associations, GLP-1RAs are being increasingly considered as a potential adjunctive option to traditional therapeutic regimens, although a definitive characterization of their impact on HS remains limited and requires further investigation. Multicenter data and proof-of concept analyses suggest that the implementation of these agents may be associated with a clinical response in approximately 60–62% of patients, although these findings are derived primarily from observational data [93,95,96,97]. Particularly noteworthy are observations of patients who, despite no modification to their primary dermatological treatment, experienced a reduction in purulent discharge, fewer exacerbations, and substantial pain relief following the introduction of GLP-1 analogues (such as liraglutide or semaglutide). In the case of liraglutide, the mean Visual Analogue Scale (VAS) score for pain decreased from 5.6 to 3.2 [93]. Studies on this agent also recorded an improvement in Hurley staging, descending from a mean of 2.6 to 1.1, while observations of patients on semaglutide revealed a progressive decline in disease activity as measured by the HASI-R scale (from 11.34 to 7.45) [98] and the HS-PGA, particularly in moderate to severe cases [99]. These findings suggest a potential adjuvant benefit of this drug class, although confirmation in controlled studies is warranted.

The therapeutic benefit of GLP-1RAs in HS is likely multifactorial and extends beyond the secondary effects of weight loss. A critical aspect highlighted in recent reports is the direct influence of semaglutide on sebaceous gland function. The expression of GLP-1Rs within tissues associated with the pilosebaceous apparatus allows for the direct inhibition of lipogenesis and a reduction in sebum secretory activity [98]. Prospective studies have demonstrated that a 24-month course of semaglutide therapy leads to a significant decrease in sebumetry parameters, falling from 186.45 µg/cm^2^ to 138.56 µg/cm^2^ [98]. Concurrently, essential immunomodulation occurs via the NF-κB pathway, resulting in the suppression of key mediators such as IL-17, IL-22, IL-23 and TNF-α [99]. This process encompasses the dampening of the systemic inflammatory axis (indicated by reductions in hs-CRP, leukocytosis, and ESR) and the modulation of Toll-like receptors (TLRs), which may contribute to a diminished inflammatory response to the bacterial flora residing within hair follicles. Furthermore, the inhibition of matrix metalloproteinases (specifically MMP-9) may support cutaneous healing and tissue remodelling [93,95,99].

While a reduction in BMI mitigates mechanical epidermal maceration and the risk of the Koebner phenomenon, clinical evidence suggests that dermatological improvement can occur in some patients independently of significant weight loss. This is evidenced by favourable clinical outcomes observed even with low-dose oral semaglutide (3 mg daily) [99]. Nevertheless, retrospective analyses confirm that patients achieving the most substantial BMI reduction (averaging −6.2 kg) frequently exhibit superior clinical responses [96]. Furthermore, long-term administration of GLP-1RAs appears to function as a biologic-sparing therapy. Data indicate that patients exposed to semaglutide or tirzepatide have a 51% lower probability of requiring the initiation of advanced biologic agents, such as adalimumab or secukinumab [100].

The convergence of these metabolic and immunological mechanisms facilitates the interruption of the “therapeutic vicious cycle” characterized of HS. Conventional weight loss through physical exertion is often contraindicated in HS patients due to friction-induced pain and subsequent maceration within skin folds. The implementation of GLP-1RAs addresses these mechanical provocateurs while simultaneously providing systemic modulation of the immune response. Consequently, this therapeutic approach leads not only to the remission of cutaneous lesions but also to significant enhancements in psychological well-being, as evidenced by improved DLQI scores and mood stabilization measured by the Beck Depression Inventory (BDI) [93,96,98].

To comprehensively evaluate the real-world efficacy of GLP-1 analogues, objective clinical parameters must be supplemented by Patient-Reported Outcomes (PROs). A cross-sectional survey conducted at the University of Pennsylvania [97] provides pivotal evidence of high patient satisfaction regarding the integration of GLP-1RAs into established treatment regimens. Although agents such as semaglutide, tirzepatide, or liraglutide were primarily prescribed for weight management or glycaemic control, 68.2% of respondents reported a distinct and palpable improvement in their HS-specific skin condition [97]. These subjective patient perceptions align closely with objective clinical data: 61.9% of participants reported a significant decrease in the frequency of painful flares, while 66.7% noted a cessation in the formation of new inflammatory lesions. The impact on daily functioning was primarily manifested through the alleviation of chronic pain (52.4%) and the mitigation of highly stigmatizing symptoms, including purulent discharge (62%), persistent pruritus (47.6%), and the malodor associated with active fistulae (42.9%) [97]. Overall, nearly 60% of surveyed patients confirmed that GLP-1RA therapy substantially reduced the extent to which the disease dictated their social and life activities.

This improvement in quality of life may translate into a measurable reduction in the burden on the healthcare system. A detailed analysis of healthcare utilization conducted in Miami demonstrated that a one-year exposure to GLP-1 agonists results in a dramatic decline in the requirement for high-acuity care. Specifically, there was a reduction in the frequency of emergency department visits and hospitalization, falling from 11.9% to a mere 3.7%, whereas these metrics remained consistently elevated (approx. 18.8%) in the matched control group [101]. Crucially, investigators emphasize the necessity of rigorous metabolic vigilance. Within the HS population, often characterized by an advanced metabolic age and compromised body composition, the aggressive use of weight-reduction dosages carries an inherent risk of lean muscle mass depletion, or sarcopenia. Consequently, an integrated therapeutic model is advocated, wherein GLP-1RA pharmacotherapy is administered alongside optimized protein intake and resistance training. Such a multi-faceted approach maximizes dermatological benefits while simultaneously safeguarding the patient’s overall metabolic integrity [101].

The most compelling evidence supporting the disease-modifying potential of this drug class stems from large-scale population analyses utilizing the TriNetX network. A landmark study involving over 13,000 patients with a 10-year longitudinal perspective demonstrated that systemic GLP-1 agonist utilization correlates with a nearly 18% reduction in the requirement for invasive and traumatizing surgical interventions, such as incision and drainage (I&D) procedures (RR = 0.821) [102]. Furthermore, users of these agents required significantly fewer systemic antibiotic courses (mean 2.78 vs. 4.11 prescriptions) and exhibited a reduced reliance on analgesics (RR = 0.843). However, these findings are observational and should be interpreted with caution, as causality cannot be established. The same cohort also demonstrated lower mortality rates and reduced progression to severe disease stages [102].

In the context of health economics and treatment escalation, GLP-1RAs emerge as a highly effective “biologic-sparing therapy”. According to cohort analysis data [100], patients with obesity and HS receiving semaglutide or tirzepatide had 51% lower odds of requiring the initiation of costly TNF-α or IL-17 inhibitors compared to matched controls. The synergy of metabolic, immunomodulatory, and systemic benefits positions this pharmacological group as a cornerstone—albeit an underutilized one—of the modern, interdisciplinary paradigm for managing HS.

Despite the documented, pleiotropic impact of GLP-1RAs on reducing HS severity, current data highlight substantial barriers to the widespread clinical adoption of this modality. It is estimated that nearly 60% of patients with inflammatory skin disease meet the eligibility criteria for GLP-1 analogue therapy, with this figure reaching a peak of 72.3% within the HS population. Regrettably, a significant therapeutic gap persists in clinical practice: only 36.4% of eligible patients receive appropriate medical counselling, and a mere one in five (22.1%) is ultimately prescribed the medication [103]. This discrepancy is particularly concerning given the proven role of these agents in arresting cutaneous disease progression [95]. The fact that nearly 25% of patients requiring metabolic intervention remain under the exclusive care of dermatological clinics imposes a new clinical responsibility upon specialists in this field [103]. Proactive identification of indications for GLP-1RA therapy in the dermatological setting is not merely an obesity intervention; it represents a critical component of a holistic therapeutic strategy aimed at modifying the natural history of HS through the systemic attenuation of inflammation [93,95,99,103].

Table 2 presents a comprehensive summary of the existing data with regard to GLP-1RAs in the management of HS. The importance of this table lies in its ability to show the multifaceted effects of GLP-1RAs on dermatological parameters, as well as their effects on various aspects of the pathophysiology of HS.

### 5.3. Atopic Dermatitis (AD)

AD is a chronic inflammatory disease of the skin characterized by periods of relapse and remission, persistent itching, typical lesion localization and early onset. Its pathogenesis involved complex interactions between genetically determined impairments in epidermal barrier structure and function, dysregulated immune and inflammatory responses, as well as infectious and environmental factors [104]. Despite a growing understanding of the pathogenesis of AD and increasing interest in the role of GLP-1RAs in inflammatory conditions, the current literature regarding their specific impact on AD remains sparse. To date, evidence is primarily limited to small cohort studies and individual case reports, and robust clinical data are lacking [102,105,106].

GLP-1RAs appear to exert a dual therapeutic effect, addressing both metabolic and immunological drivers of the disease. On a metabolic level, they significantly mitigate IR. Simultaneously, they modulate immune dysregulation by attenuating Th2 and Th-17-mediated inflammation, which are predominantly responsible for pruritus and erythema [107,108]. Molecular mechanisms underlying these effects include the inhibition of the NF-κB pathway, a reduction in reactive oxygen species (ROS) production, the promotion of Treg expansion, and a decrease in systemic pro-inflammatory cytokines such as IL-6 and TNF-α [108,109]. Furthermore, preliminary data suggest that GLP-1R activation on eosinophils may lead to downregulation of interleukin-4 (IL-4) and interleukin-13 (IL-13), cytokines that are typically elevated in AD patients. Consequently, these AD-related biomarkers may serve as predictors for the clinical efficacy of GLP-1RA therapy [105].

Importantly, treatment with GLP-1 analogues has been associated with changes in gut microbiota composition, including an increased abundance of bacteria producing short-chain fatty acids. This shift is associated with the activation of PPAR-γ and aryl hydrocarbon receptors, which in turn upregulates the expression of filaggrin and tight junction proteins, specifically claudin and occludin. These processes facilitate the restoration of the hydrolipid barrier and a subsequent reduction in transepidermal water loss (TEWL). Moreover, obesity—often the primary indication for initiating GLP-1RA treatment—is a known driver of intestinal permeability. This “leaky gut” allows LPS toxins to enter the bloodstream, thereby exacerbating AD symptoms. By improving intestinal barrier integrity, GLP-1RAs may help reduce systemic inflammation that triggers disease flares [108]. It is therefore vital to emphasize that obese patients with AD may derive benefits from GLP-1RA therapy that exceed those of standard biological treatments, as these agents address the metabolic underpinnings of the disease that conventional dermatological therapies fail to correct [105,107,108,109].

Research involving patients with Class I, II, III obesity indicates that improved glycaemic control, coupled with weight reduction, may decrease the necessity for both systemic and topical glucocorticosteroids, ultimately alleviating the clinical manifestations of AD [109].

Further evidence of this potential is found in a study of patients with chronic spontaneous urticaria (CSU). Following the introduction of GLP-1RA therapy, a rapid and significant decrease in urticarial activity was observed as early as the third week of treatment, occurring alongside weight loss and stabilized glucose levels. Remission was maintained even as standard CSU treatments were tapered [107]. This clinical observation supports the hypothesis that GLP-1 analogues may elevate the mast cell degranulation threshold while inhibiting NF-κB and TNF-α signalling pathways. Given that the restoration of the balance between pro- and anti-inflammatory responses is a fundamental therapeutic goal in both AD and CSU, it stands to reason that these agents could represent a highly effective adjunctive therapy for refractory AD in patients presenting with T2DM and/or metabolic disorders. However, extrapolation of CSU data to AD should be made with caution due to differences in pathophysiology.

Table 3 provides a summary of the clinical evidence for the use of GLP-1RAs in AD and related eosinophil- and mast cell-mediated skin diseases, including CSU. It incorporates large-scale cohort studies and case reports to highlight their effect on proxies of disease severity, cardiovascular risk factors, and symptom control. Furthermore, it also includes proposed immunomodulatory mechanisms of action for these drugs, such as the regulation of Th2 responses (IL-4, IL-13), NF-κB pathways, eosinophils, and mast cells, thereby highlighting the potential role of GLP-1RAs in type 2 inflammatory skin diseases.

### 5.4. Alopecia

Current clinical data regarding the relationship between GLP-1RAs and alopecia remain multifaceted and, at times, contradictory, largely depending on the specific clinical subtype. While some researchers report significant improvements in hair density [110,111,112] and a decreased reliance on conventional hair loss therapies [113], others express concern over a potential risk of hair loss following the initiation of GLP-1RA treatment [114,115,116,117]. Although a hypothesis regarding the risk of hair shedding linked to GLP-1 analogues has been proposed, emerging evidence suggests that the actual clinical significance of this phenomenon remains uncertain and may be less substantial than initially suggested [106].

Central Centrifugal Alopecia (CCCA) is a progressive, scarring form of permanent hair loss that typically originates at the vertex and expands peripherally. Predominantly affecting women of African descent, it is characterized by follicular fibrosis [118]. Interestingly, the severity of T2DM has been mechanistically linked to the pathogenesis of CCCA [110]. Optimal metabolic control, evidenced by a reduction in glycated hemoglobin (HbA1c) alongside standard CCCA treatments, has been associated with improved clinical outcomes. This synergy likely stems from a stabilized metabolic state, which attenuates the pro-inflammatory and fibrotic environment driving follicular destruction [110].

Folliculitis Decalvans (FD) represents another chronic inflammatory alopecia, marked by recurrent flares and scarring [119]. Its pathogenesis involves a complex interplay between immune dysregulation and microbial factors leading to chronic inflammation and irreversible hair loss. A notable case involves a male patient with refractory FD who was treated with tirzepatide while concurrently using topical clobetasol and crisaborole. Although previous topical therapies had proven ineffective, the introduction of tirzepatide led to a significant reduction in pain, exudate, and disease flares. Physical examination revealed a marked decrease in erythema and, crucially, visible hair regrowth- a finding particularly significant in scarring alopecia. During treatment, the patient achieved a weight loss of approximately 22.7 kg (50 lbs), which improved insulin sensitivity and suppressed the chronic inflammatory pathways involved in FD. Notably, tapering the tirzepatide dose led to a resurgence of FD symptoms, suggesting a possible association rather than confirming a direct causal effect [111]. Similarly, a case report of a patient with androgenetic alopecia (AGA) treated with tirzepatide documented a weight loss of 13.6 kg (30 lbs) accompanied by substantial hair thickening [112]. The authors suggest that enhanced insulin sensitivity may contribute to mitigating follicular miniaturization by improving microcirculation and potentially downregulating 5-α-reductase activity.

Despite these promising therapeutic observations, the literature also provides evidence of adverse hair loss events following treatment initiation [114,116]. Retrospective cohort studies utilizing the TriNetX database analysed patients diagnosed with telegenic effluvium (TE), anagen effluvium (AE), alopecia areata (AA), androgenetic alopecia (AGA), and other non-scarring hair loss (ONSH) patterns following semaglutide or tirzepatide use [114,116]. While TE and AE were diagnosed within one year of treatment, AA, AGA, and ONSH were frequently identified as early as six months. Researchers emphasized that BMI changes were relatively modest and comparable across groups, suggesting that these findings cannot be solely attributed to rapid or extreme weight loss [114].

Another TriNetX-based study focused on T2DM patients examined the risk of AGA following therapy with tirzepatide, liraglutide, dulaglutide, and semaglutide. All four agents were associated with an increased risk of AGA, with tirzepatide showing the highest correlation. The risk was progressively lower for semaglutide and dulaglutide, with liraglutide demonstrating the weakest association [116]. Paradoxically, tirzepatide was also linked to the most significant reductions in HbA1c and improvements in insulin sensitivity. This phenomenon has been hypothesized to be related to an increase in insulin-like growth factor-1 (IGF-1) levels, which can enhance androgen receptor activity and accelerate AGA progression. This suggests that rapid metabolic optimization may, in some cases, unmask a genetic predisposition to AGA. However, the risk associated with elevated IGF-1 must be balanced against the systemic benefits of reduced chronic inflammation resulting from improved insulin sensitivity [116].

Data also indicate a significant correlation between hair loss and the diagnosis of T2DM itself. Reports of hair loss were more frequent among patients using GLP-1 analogues for T2DM management compared to those using the same medications solely for weight reduction [115]. This supports the hypothesis that the chronic inflammation and vascular microdamage associated with diabetes serve as predisposing factors for hair loss, which could be influenced by metabolic changes during therapy. Furthermore, the T2DM demographic is generally older, a factor naturally associated with physiological hair thinning and reduced follicular reserve. Consequently, while reporting rates are higher, they do not necessarily exceed statistical expectations or imply a direct toxic effect of the drug on the hair follicle. Instead, this evidence suggests that hair loss may be secondary to metabolic shifts and weight reduction rather than a direct pharmacological effect, although causality cannot be definitively established.

A pronounced female predominance in reporting is also evident, likely due to a higher sensitivity of the female hormonal profile to rapid metabolic changes [120], as well as a greater propensity among women to report adverse dermatological effects (reporting bias) [121].

In conclusion, while current findings are encouraging, most available data are derived from isolated case reports and retrospective database analyses. The overall level of evidence remains limited, and there is a need for longitudinal observation and prospective clinical trials that account for gender differences and specific alopecia phenotypes to further clarify the role of GLP-1RAs in hair disorders.

Table 4 shows an overview of the available literature on the use of GLP-1RAs for hair disorders, including favourable and adverse effects of the therapy on different subtypes of alopecia. It has been compiled by incorporating real-life data, pharmacovigilance studies, and case-based experiences of the therapy by considering the intricate relationship between metabolism, hormonal balance, and pro-inflammatory pathways influencing the therapeutic effects of the therapy.

### 5.5. Limitations of Current Evidence

Current evidence on the use of GLP-1 analogues in skin conditions, including psoriasis, HS, AD, and alopecia, is limited by several methodological constraints. Most studies involved relatively small sample sizes, often encompassing only a few patients or small cohorts (*n* = 3–24 for psoriasis [9,15,26,52,82,85,87,91], *n* = 5–20 for HS [21,22,23,24,25,26,27,28,29,30], *n* = 2–10 for AD [105,109], *n* = 1–10 for alopecia [106,110,111,112,113,114,115,116,117]), which restricts the generalizability of the results.

A substantial number of studies lacked control groups or randomization. Many were conducted as open-label, retrospective, or single-arm investigations, limiting casual inference and the ability to account for placebo effects [49,86,87,91,92,93,95,96,98,103,106,110,111,112,113,114,115,116,117]. Furthermore, research conducted at single centres may not fully capture the variability present in larger, more diverse patient populations [93,97,99,100,101,102,103,105].

The follow-up periods were often short, ranging from a few weeks to several months, which hampers the assessment of long-term durability of treatment effects and safety in chronic or relapsing disorders [26,52,82,85,95,101,102,112,114]. Many studies also lacked standardized clinical measures and objective markers of disease severity. Instead, outcomes frequently relied on patient-reported assessments, retrospective PASI, Hurley, or EASI scores, diagnostic coding, medical record reviews, prescriptions, or unverified sources [9,26,38,81,87,100,103,105,106,109,110,114,115,117].

Additional limitations included the absence of biological data (e.g., cytokine levels, GLP-1, HbA1c), lack of control over concomitant therapies, inter-center variability, and potential confounding factors such as diet, physical activity, or stress [38,84,92,95,96,106,110,111,112,114,115,116]. In vitro studies, where applied did not always replicate the complex cellular interactions occurring in vivo, and drug concentrations used in laboratory experiments may not correspond to clinically achievable levels [38]. For studies on alopecia, evaluating treatment effects was particularly challenging due to the absence of trichoscopic or clinical assessments of hair loss severity, sex-specific differences in pathophysiology, and potential confounding effects of weight loss [106,110,111,112,113,114,115,116,117].

In summary, while preliminary findings suggest possible therapeutic benefits of GLP-1 analogues in skin disease, the current evidence base remains limited, and there is a need for larger, prospective, RCTs conducted across multiple centers. Such studies should include appropriate control groups, standardized clinical and biomarker assessments, and longer follow-up periods to more accurately evaluate both the efficacy and safety of these therapies.

## 6. Pharmacokinetic and Formulation Considerations Relevant to Dermatological Applications

GLP-1RAs are peptide-based therapeutics characterized by unfavourable physicochemical properties for non-parenteral delivery, including high molecular weight, hydrophilicity, and low membrane permeability, which limit passive diffusion across biological barriers [122]. A major limitation of native GLP-1 is its extremely short half-life (~2 min), resulting from rapid enzymatic degradation by DPP-4 and renal clearance [123]. These early pharmacokinetic limitations were a major barrier in the initial clinical development of GLP-1-based therapies, necessitating the design of more stable and longer-acting analogues [124]. To overcome these constraints, multiple structural modification strategies have been employed, including amino acid substitutions to enhance enzymatic stability, fatty acid conjugation enabling reversible albumin binding, and fusion to large protein domains such as Fc fragments [125,126,127]. For instance, lipidation not only delays renal clearance but also prolongs systemic exposure through albumin binding, representing a key milestone in the evolution of GLP-1 therapeutics [128]. These modifications reduce renal elimination and proteolytic degradation, thereby significantly prolonging systemic exposure.

As a result, currently available GLP-1RAs exhibit heterogeneous pharmacokinetic profiles determined by their molecular architecture. Short-acting agents such as exenatide require frequent dosing due to rapid clearance [129], whereas long-acting analogues, including liraglutide, semaglutide, dulaglutide, and tirzepatide, demonstrate prolonged half-lives ranging from approximately 13 h to several days, supporting once-daily or once-weekly administration [130,131,132,133]. This progressive extension of half-life reflects a broader trend in GLP-1 drug development toward improving patient adherence and therapeutic durability through less frequent dosing schedules [134]. These extended profiles are closely linked to albumin binding and reduced renal filtration, as well as delayed absorption kinetics and prolonged time to maximum concentration (Tmax), which may additionally contribute to improved tolerability [134]. Importantly, pharmacokinetic variability may also influence the safety profile, as prolonged exposure has been associated with an increased risk of dose-dependent adverse events [123].

Formulation strategies have played a critical role in optimizing the pharmacokinetic behaviour of GLP-1RAs. For instance, extended-release exenatide utilizes poly-(D,L-lactide-co-glycolide) microsphere encapsulation, enabling gradual drug release over several weeks [135]. Another important advancement is the development of oral semaglutide, co-formulated with the absorption enhancer SNAC, which facilitates transcellular absorption across the gastric mucosa and protects the peptide from enzymatic degradation [136,137]. This approach illustrates ongoing efforts to diversify routes of administration for peptide-based therapeutics while maintaining adequate systemic exposure [137]. Despite this innovation, oral bioavailability remains low (~0.8%), reflecting the inherent limitations of peptide delivery across epithelial barriers [138].

In addition to formulation challenges, GLP-1RAs exhibit specific pharmacokinetic interaction profiles. Clinically significant interactions mediated by drug-metabolizing enzymes or transporters are generally not observed; however, mechanism-based interactions related to delayed gastric emptying may alter the absorption of concomitant medications, including oral contraceptives and levothyroxine, necessitating clinical monitoring in selected cases [123]. Furthermore, changes in body composition, renal function, or metabolic status may influence drug distribution and clearance, although these mechanisms remain incompletely understood [123].

These physicochemical, pharmacokinetic, and formulation-related constraints are particularly relevant when considering dermatological applications. The large molecular size and hydrophilic nature of GLP-1RAs inherently limit their penetration through the stratum corneum, posing a significant barrier to topical delivery. Consequently, current therapeutic approaches rely on systemic administration, which may not allow for targeted cutaneous drug distribution. From a translational perspective, future development of GLP-1-based therapies may benefit from strategies aimed at improving tissue-specific delivery while preserving the favourable systemic immunometabolic effects observed with current formulations [134]. Although the immunometabolic effects of GLP-1RAs are promising in inflammatory skin diseases, further research into optimized delivery systems and formulation strategies will be necessary to enhance tissue-specific efficacy and fully realize their translational potential in dermatology.

## 7. Safety Profile and Dermatologic Adverse Effects of GLP-1RAs

GLP-1RAs provide well-established metabolic and cardiovascular benefits, including significant weight reduction, improved glycaemic control, and cardiovascular risk reduction. Current research also reports broader therapeutic effects across multiple organ systems, as well as potential reductions in all-cause mortality in selected populations [139,140]. Their expanding clinical use, however, has also been associated with a range of adverse effects, including those relevant to dermatology [140,141].

GLP-1RAs primarily cause gastrointestinal and cutaneous side effects. Less commonly reported events include neurological symptoms such as headache and dizziness, with rare psychiatric manifestations, as well as hypoglycaemia, particularly when used in combination with insulin or sulfonylureas. Additional effects may include mild increases in heart rate and weight loss-related structural changes [142].

The most common side effects associated with GLP-1RAs are gastrointestinal disturbances, including nausea, vomiting, diarrhoea, and constipation [143,144]. These effects are typically dose-dependent and most frequent during the early phases of treatment and dose titration, while their frequency and severity vary across different agents and patient populations [143,145]. Despite their high prevalence, they are generally mild to moderate in severity and tend to diminish over time, yet they remain a leading cause of treatment discontinuation [145].

While emerging research suggests anti-inflammatory and immunomodulatory effects of GLP-1RAs that may benefit dermatologic conditions, their potential to cause cutaneous adverse effects should also be considered. Cutaneous reactions are less frequently reported but are of increasing relevance in dermatologic practice. Dermal hypersensitivity including injection-site reactions, such as erythema, pruritus, and localized rash or itching, remain the most described dermatologic manifestations. They are typically mild, transient, or otherwise manageable, and therefore rarely lead to treatment discontinuation [40,141,142]. Research also suggests that GLP-1RAs may be associated with a broader spectrum of cutaneous adverse events, including urticaria, angioedema, and generalized hypersensitivity reactions, as well as rare immune-mediated conditions such as bullous pemphigoid and pemphigus vulgaris [146,147,148,149,150]. However, the overall strength of evidence concerning the rarer side effects remains limited, as most data are derived from case reports.

An emerging area of interest is the impact of rapid weight loss induced by GLP-1RAs on skin structure, appearance, and facial aging. Significant reductions in subcutaneous adipose tissue may lead to decreased facial volume, increased skin laxity, and changes in contour, which reflect the structural role of adipose tissue in maintaining skin support and integrity [151,152]. This phenomenon is often colloquially described as “Ozempic face”, referring to the appearance of facial hollowing, sagging and volume loss, which is becoming a growing concern among Ozempic patients [153,154]. While “Ozempic face” is not a formal medical diagnosis, it highlights the aesthetic consequences of significant adipose tissue loss, becoming increasingly relevant not only to dermatologists but also to plastic surgeons and aesthetic practitioners [155,156]. However, it should be noted that such changes in facial appearance are primarily related to rapid weight loss itself and are not specific to GLP-1RAs therapy [51,157].

Aside from cosmetic effects, the loss of adipose tissue may have functional implications for the skin. Adipose tissue is increasingly recognized as an active immunometabolic organ involved in cutaneous immune regulation, antimicrobial defense, and wound healing. Adipocytes contribute to immune cell activation, cytokine signalling, and tissue repair processes through the secretion of antimicrobial peptides, adipokines, and interactions with immune cells [78,79,158]. Because of its role in maintaining dermal structure and skin homeostasis, rapid reduction in adipose tissue may theoretically disrupt local tissue function. This represents a potential concern, particularly in patients with chronic inflammatory dermatoses, and highlights the need for further investigation.

In summary, while GLP-1RAs provide well-established systemic benefits, their use is associated with a range of adverse effects, including relatively common cutaneous reactions. Although these are usually mild and manageable, clinicians should remain aware of their occurrence for prompt diagnosis and individualized treatment, or, in some cases, GLP-1RAs treatment discontinuation. Furthermore, the rising recognition of the undesirable facial changes, often referred to as “Ozempic face”, may pose a future challenge for both dermatologists and plastic surgeons, which warrants further consideration and establishing reliable treatment strategies. Additional studies are needed to better understand the underlying cellular mechanisms of GLP-1RA-related skin effects and their clinical implications.

## 8. Conclusions

The GLP-1R signalling pathway is thought to represent a multifaceted regulatory mechanism potentially involved in various aspects of skin biology. This includes keratinocyte function, hair follicle biology, and immune cell function in relation to immunometabolic signalling. The expression of GLP-1R in skin structures suggests the presence of a signalling axis that may be relevant for various cell functions, including cell proliferation, migration, differentiation, and remodelling. GLP-1R signalling has been shown in experimental models to activate various cell signalling cascades, including PI3K/Akt, MAPK/ERK, cAMP/PKA/CREB, and AMPK, which may contribute to cytoprotective, regenerative, and anti-inflammatory activities, potentially supporting tissue homeostasis. Moreover, its role in modulating immune responses, such as the inhibition of NF-κB-dependent inflammatory responses, iNKT cell function, and IL-17 signalling pathways, suggests a possible role in chronic inflammatory skin diseases. Additionally, its involvement in angiogenesis, oxidative stress, and macrophage polarization points to a potential role in wound healing processes. In conclusion, GLP-1R signalling appears to represent a potential interface between metabolism and skin biology, supporting the hypothesis of a possible therapeutic role of GLP-1RAs. However, despite promising preliminary findings, particularly in psoriasis, the current clinical evidence remains limited and is largely based on small studies and observational data. Therefore, further well-designed, large-scale clinical trials are required to confirm the magnitude, consistency, and clinical relevance of these effects.

## Figures and Tables

**Table 1:** Summary of clinical and mechanistic effects of GLP-1 receptor agonists (GLP-1RAs) in psoriasis [5,9,15,26,38,49,52,80,81,82,84,85,86,87,88,89,90,91,92].

Study	Study Design	Population	Intervention	Control	Endpoints
Schwandt et al., 2015 [80]	Prospective, multicenter	*n* = 222,078 T2DM patients (232 with concomitant PSO)	NA (Observational)	T2DM without psoriasis	Metabolic/clinical parameters, hospitalizations, T2DM control
Reid et al., 2013 [91]	Case report	54-year-old obese male (BMI 42.1)	Liraglutide (up to 3 mg) + Acitretin	NA	PASI, DLQI, weight, BMI, insulin resistance
Cheng et al., 2026 [81]	Retrospective cohort study	*n* = 20,349	SGLT2i	DPP4i or GLP1RA groups	Adverse outcomes
Lin et al., 2022 [15]	RCT	*n* = 24 (PSO + T2D)	Liraglutide + Acitretin	Acitretin + placebo	PASI, DLQI, BMI, waist circumference, HbA1c, HOMA-IR, C-peptide, lipids, skin inflammation markers (IL-17, IL-23, TNF-α)
Petković-Dabić et al., 2025 [92]	Open-label, RCT	*n* = 55 (obese patients with PSO & T2DM)	Semaglutide (1.0 mg/week)	Metformin	PASI, DLQI, metabolic parameters (LDL-C, weight)
Ching et al., 2025 [5]	Retrospective cohort study	*n* = 74,910 (T2DM patients)	GLP-1RAs (albiglutide, dulaglutide, exenatide, liraglutide, lixisenatide, semaglutide)	Patients without GLP-1RA exposure	Incidence of psoriasis and HS
Hogan et al., 2011 [26]	Case series & in vitro experiment	*n* = 3 (obese patients with T2DM & PSO)	Exenatide or Liraglutide	NA	PASI, pruritus, sleep quality, iNKT cell counts in circulation and psoriatic plaques, cytokine production
Yang et al., 2019 [38]	in vitro study	HaCaT keratinocytes	Liraglutide ± LPS	MEM only or LPS 150 ng/mL	Cell viability, TNF-α, IL-6, p-NF-κB p65, p-STAT3, macrophage migration
Veale et al., 2025 [84]	Serum biomarker analysis	*n* = 171 (RA:74, PsA:97)	Observational	HC (*n* = 15)	Serum levels of CRP, SAA, sICAM-1, sVCAM-1, MMPs, GLP-1, GIP-1, insulin, PP, leptin, C-peptide
Faurschou et al., 2013 [9]	Translational/biopsy-based study	*n* = 6	Biopsy analysis	Healthy skin and non-lesional psoriatic skin	GLP-1R expression (skin and blood), IL-17, TNF-α
Ahern et al., 2013 [52]	Prospective, open-label cohort study	*n* = 7 patients with chronic plaque psoriasis, obesity, and T2DM	Liraglutide (0.6 mg to 1.2 mg daily)	None (baseline as control)	PASI, DLQI, body weight, fasting glucose, circulating iNKT cells, monocytes producing TNF-α
Lee et al., 2025 [89]	Retrospective cohort (Real-world)	Adults ≥ 18 years with T2DM	GLP-1RAs (semaglutide, liraglutide, dulaglutide)	DPP-4i	Incidence of autoimmune diseases
Faurschou et al., 2014 [49]	Case report	59-year-old male (T2DM + Psoriasis)	Liraglutide (up to 1.2 mg/day)	NA	PGA, HbA1c, weight
Xu et al., 2019 [82]	Prospective cohort study	PsO and T2DM (*n* = 7)	Liraglutide (up to 1.8 mg/day)	Baseline values	PASI, DLQI, BMI, waist circumference, HbA1c, fasting C-peptide, HOMA-IR, LDL, CRP, skin histopathology
Faurschou et al., 2015 [85]	Randomized, placebo-controlled trial	Obese, glucose-tolerant patients with plaque PsO (*n* = 20)	Liraglutide (titrated to 1.8 mg/day)	Placebo injection	PASI, DLQI; bodyweight, cholesterol, hsCRP
Nicolau et al., 2026 [86]	Prospective, open-label cohort study	PsO and obesity, non-diabetic (*n* = 43)	Semaglutide (dose titration per standard protocol)	Baseline values	PASI, DLQI, pain, depressive symptoms, body weight, metabolic and inflammatory markers
Buysschaert et al., 2012 [87]	Case report	61-year-old male (T2DM + PsO)	Exenatide (2 × 5 µg/day)	None	PASI, weight, HbA1c, US-CRP
Costanzo et al., 2021 [88]	Case report	73-year-old male (T2DM + severe PsO)	Semaglutide (titrated to 1 mg/week)	None	PASI, DLQI; HbA1c, fasting glucose, BMI
Nowowiejska et al., 2023 [90]	Case report	34-year-old woman with mild psoriasis and IR	Liraglutide	None	Clinical observation of skin lesions

Abbreviations: T2DM—type 2 diabetes mellitus; PSO/PsO—psoriasis; PsA—psoriatic arthritis; RCT—randomized controlled trial; NA—not applicable; BMI—body mass index; PASI—Psoriasis Area and Severity Index; DLQI—Dermatology Life Quality Index; SGLT2i—sodium-glucose cotransporter-2 inhibitors; DPP4i/DPP-4i—dipeptidyl peptidase-4 inhibitors; GLP-1RA/GLP-1RAs—glucagon-like peptide-1 receptor agonist(s); HbA1c—glycated hemoglobin; HOMA-IR—homeostatic model assessment of insulin resistance; LDL-C—low-density lipoprotein cholesterol; LDL—low-density lipoprotein; HS—hidradenitis suppurativa; iNKT cells—invariant natural killer T cells; TNF-α—tumor necrosis factor alpha; IL—interleukin; LPS—lipopolysaccharide; p-NF-κB p65—phosphorylated nuclear factor kappa B p65 subunit; p-STAT3—phosphorylated signal transducer and activator of transcription 3; HaCaT keratinocytes—immortalized human keratinocyte cell line; RA—rheumatoid arthritis; HC—healthy controls; CRP—C-reactive protein; hsCRP—high-sensitivity C-reactive protein; US-CRP—ultrasensitive C-reactive protein; SAA—serum amyloid A; sICAM-1—soluble intercellular adhesion molecule-1; sVCAM-1—soluble vascular cell adhesion molecule-1; MMPs—matrix metalloproteinases; GLP-1R—glucagon-like peptide-1 receptor; GIP-1—glucose-dependent insulinotropic polypeptide-1; PP—pancreatic polypeptide; PGA—Physician Global Assessment.

**Table 2:** Summary of clinical and mechanistic effects of GLP-1 receptor agonists (GLP-1RAs) in hidradenitis suppurativa (HS) [93,95,96,97,98,99,100,101,102,103].

Study	Study Design	Population	Intervention	Endpoints
Gouvrion et al., 2025 [95]	Retrospective multicenter cohort	*n* = 66; BMI 39.4; 86% T2DM	GLP-1RA (semaglutide, dulaglutide, liraglutide)	HS-PGA, flares, pain, suppuration, DLQI
Granovsky et al., 2026 [103]	Retrospective multicenter cohort	*n* = 346; BMI > 30	GLP-1RA eligibility	Prescription and counseling rates
Nicolau et al., 2024 [93]	Retrospective chart review	*n* = 14; BMI 39.3 ± 6.2	Liraglutide 3 mg	Hurley stage, pain, hs-CRP, QoL, mood
Encarnacion et al., 2026 [96]	Retrospective cohort	*n* = 142; overweight/obese	GLP-1RA	HS activity, BMI, HbA1c
Jabin et al., 2026 [98]	Prospective observational	*n* = 110; age 18–65	Semaglutide	HS activity (HASI-R), acne IGA, sebum production
Gupta et al., 2025 [97]	Prospective observational	*n* = 22; 91% female	GLP-1RA	Patient-reported HS severity, QoL, flares, pain, drainage, odor, pruritus
Lam et al., 2026 [101]	Matched-pairs retrospective	*n* = 78 matched pairs; 81% female; BMI 38.5	GLP-1RA	Healthcare utilization (HU), BMI, HS severity
Kasheri et al., 2026 [100]	Retrospective TriNetX cohort	*n* = 1351 per cohort; BMI ≥ 30	GLP-1RA	Biologic therapy initiation
Islam et al., 2026 [99]	Retrospective chart review	*n* = 40; BMI 38.8 ± 7.2; 80% diabetes	GLP-1RA	HS-PGA, pain, leukocyte count, ESR, CRP, IL-6, BMI, HbA1c
Fite et al., 2026 [102]	Retrospective TriNetX	*n* = 11,950 per cohort	GLP-1RA	ER visits, antibiotic use, analgesic use, incision/drainage, biologics, mortality

**Table 3:** GLP-1 Receptor Agonists (GLP-1RAs) in Atopic Dermatitis (AD) and Eosinophil- and Mast Cell-Mediated Skin Diseases [105,107,109].

Section/Author, Year	Study Type	Population/*n*	Intervention	Control	Endpoints
Atopic Dermatitis (AD)					
Braun et al., 2025 [105]	Retrospective cohort analysis (TriNetX)	T2DM + AD (*n* = 17,099 pairs after PSM)	GLP-1RA	T2DM patients without AD	Major adverse cardiovascular events (MACE)
Burke et al., 2026 [109]	Retrospective cohort study (TriNetX US)	Obesity class I–III and AD (*n* = 7239)	GLP-1RA	Patients with same obesity class, no GLP-1RA	Systemic and topical corticosteroid use
**Chronic Spontaneous Urticaria (CSU)/Other Mast Cell/Eosinophil-Dependent Conditions**					
Kwiek et al., 2026 [107]	Case report	2 female patients (CSU onset 2019 and 2020)	Semaglutide/Tirzepatide	none	UAS7, angioedema

**Table 4:** Effects of GLP-1 receptor agonists (GLP-1RAs) on hair disorders: clinical evidence, safety signals, and mechanistic insights [106,110,111,112,113,114,115,116,117].

Study	Design	Population	Exposure	Comparator	Outcomes
Herrera & Bordeaux, 2026 [114]	Retrospective cohort	≥18 yrs; *n* = 576,250 per cohort	Semaglutide, tirzepatide	Metformin	TE, AE, AA, AGA, ONSH
Burke et al., 2025 [106]	Retrospective cohort	*n* = 283	GLP-1RA	None	AGA, TE
Daniel et al., 2025 [115]	FAERS analysis	*n* = 220,872 AE reports	Multiple GLP-1RA	None	Cutaneous adverse events
Desir et al., 2025 [110]	Retrospective study	CCCA; *n* = 81	GLP-1RA + standard therapy	None	Clinical response
Singal et al., 2025 [116]	Retrospective cohort	Semaglutide *n* = 586,157, Dulaglutide *n* = 451,773, Liraglutide *n* = 213,012, Tirzepatide *n* = 198,269	GLP-1RA class	Metformin	AGA
Nakhla et al., 2025 [117]	Pharmacovigilance	FAERS *n* = 227,397 reports	GLP-1RA	Other anti-diabetic drugs	Hair loss
Hill et al., 2026 [113]	Retrospective cohort	Scarring alopecias; *n* = 1171 per cohort	Semaglutide, tirzepatide	Matched cohort	HRU
Morrissette et al., 2024 [111]	Case report	1 patient, recalcitrant FD	Tirzepatide	None	Clinical improvement
Gordon et al., 2024 [112]	Case report	1 patient, AGA with insulin resistance	Tirzepatide	None	Hair regrowth, hair density

## Data Availability

No new data were created or analyzed in this study. Data sharing is not applicable.
